# Supplementary material for: Occurrence of Escherichia coli Pathotypes in Diarrheic Calves in a Low-Income Setting
Source: Pathogens. 2022 Dec 27;12(1):42. doi: 10.3390/pathogens12010042 (PMC9861035; doi:10.3390/pathogens12010042)
Supplement: Supplementary file 1 [file pathogens-12-00042-s001.zip › pathogens-2085345-supplementary.pdf]

Supplementary Table S1. The background information of the 100 diarrheic calves including the breed, feed, and housing practices in 98 farms in the Basona Werana district, Ethiopia. wks; weeks; NS: not started

Farm Description				
**Diarrheic calf information**	**Category**		**Diarrheic calves -n(%)**	**Total**
Which sex the calf is?	Sex	Male	47 (47)	100
		Female	53 (53)	
How old the calf is?	Age (weeks)	(0 -2] wks	3 (3)	100
		(2 – 4] wks	30 (30)	
		(4 – 7] wks	27 (27)	
		(7 – 10] wks	39 (39)	
What is the breed of the calf?	Breed	Indigenous-breed	15 (15)	100
		Cross-breed	85 (85)	
Did you feed colostrum for the calf?	Colostrum_feeding	No	11 (11)	100
		Yes	89 (89)	
If yes to the above question when was the first colostrum feeding?	Clostrum_feeding_time	(0 – 6] hrs	43 (43)	100
		(6 – 24] hrs	46 (46)	
How did you feed colostrum to the calf?	Colostrum_feeding_method	Suckle	72 (72)	100
		Hand	17 (17)	
What are your supplementary feed choices?	Supplementary_feed	NS	16 (16)	100
		Grazing	27 (27)	
		Concentrates	9 (9)	
		Hay	23 (23)	
		Combined	25 (25)	
**Calving house**	**Category**		**Farms n (%)**	
What is the calving housing at your farm?	Calving_house	Pen	68 (69)	98
		Barn	30 (31)	
What is the nature of the floor for calving house at your farm?	Calvin_house_floor	Soil	72 (74)	98
		Concret	8 (8)	
		Stonelined	18 (18)	
How cleaned is the calving house?	Calving_house_cleaness	Very poor	17 (17)	98
		Poor	61 (63)	
		Good	15 (15)	
		Very good	5 (5)	

n & %: number and percentage of diarrheic calves/farms, Wks – weeks, hrs – hours, NS – not started

Supplementary Table S2. Calving house hygiene assessment checklist

No	Calving House hygiene checklist	Yes ☒	No ☒	Points	Hygienic Rank
1.	Proper manure disposal - no manure leftovers	1	0	≤ 1	Very poor
2.	Properly dried – no wet	1	0	2	Poor
3.	Presence of air circulation	1	0	3	Good
4.	Absence of other nearby wastes	1	0	4	Very good

Supplementary Table S3. The table shows farm descriptions by the number of animals, farm types, and subdistricts. A total of 98 farms of three types; entrepreprise, family, and research were sampled in 10 subdistricts in Basona Werana district, Ethiopia. The farms were categorized into two sizes, small farm (SF), 2- 6 animals per farm, and medium farm (MF), 7-3 animals per farm with an average and median values of 6.5 and 6 animals per farm.

		Number of animals				Farm size		
						SF	MF	
		2 - 6	7 - 32	Mean	Median	n		Total
**Farm type**	Enterpreneur	0	2					
	Family	51	44	6.5	6	51	47	98
	Research	0	1					
**Subdistrict**	Angolela	23	24					
	Keyet	4	1					
	Bakelo	6	8					
	Chacha	1	0					
	Wushawushegn	3	0	6.5	6	51	47	98
	Sariya	6	6					
	Debre Birhan	4	2					
	Abamote	1	1					
	Weyeniyana	1	2					
	Korma Gefiya	2	3					

n - number of farms in each category, SF – small farm, MF – medium farm
